# Long-term effects of doping with anabolic steroids during adolescence on physical and mental health

**DOI:** 10.1007/s00132-024-04498-3

**Published:** 2024-04-23

**Authors:** K. Berger, F. Schiefner, M. Rudolf, F. Awiszus, F. Junne, M. Vogel, C. H. Lohmann

**Affiliations:** 1 Department of Orthopaedic Surgery, Otto-von-Guericke University, Leipziger Str. 44, 39120 Magdeburg, Germany; 2 Department for Psychosomatic and Psychotherapy, Otto-von-Guericke University, Magdeburg, Germany

**Keywords:** Doping substances, Competitve sports, Young athletes, Psychological, State driven, Dopingsubstanzen, Leistungssport, Jugendliche Athleten, Psychologisch, Staatlich gesteuert

## Abstract

**Background:**

Systematic doping programs like in the GDR were applied in adolescent competitive athletes to induce supramaximal athletic performance. The substances had adverse somatic and psychological effects. The psychological development of the young athletes was impaired and they suffered in adulthood from long-term effects and secondary diseases even years after the doping period.

**Method:**

The study compared three groups: competitive athletes with doping (I), competitive athletes without doping (II) and persons with no sports activities (III). Somatic and psychological diseases were analyzed to identify the adverse effects of doping in the most vulnerable phase of development in adolescence. Participants were asked to supply a patient history and completed a questionnaire with standardized psychological tests.

**Results:**

The doping cohort had a higher rate of somatic diseases, psychological disorders and social and professional difficulties. The differences were gender–specific with males more often having impaired liver function, depression, tumors and difficulties associated with the workplace .

The doping group reported more emotional and physical neglect during childhood. They proved to be less optimistic but more pessimistic, to perceive less social support and to be more depressive. The study identified less extraversion and more neuroticism. Posttraumatic stress disorder (PTSD) occurred in a small number of participants in the doping group. Doping is associated with psychiatric variables. Predictors were the subscale identifying feelings of the Toronto alexithymia scale 20 (TAS-20), the sense of coherence and the Beck depression inventory 2 (BDI-II) and the Beck depression inventory (BDI).

**Conclusion:**

Physical and psychosocial effects imply correlation with the application of doping substances but might not only be due to the side effects of these substances but also caused by the system, which exerts great psychological pressure and stress during adolescence, a highly vulnerable phase.

## Abstract (de)

**Hintergrund:**

Systematische Dopingprogramme wie beispielweise in der DDR wurden bei jugendlichen Leistungssportlern eingesetzt, um supramaximale sportliche Leistungen zu erzielen. Die Substanzen hatten nachteilige Effekte auf Körper und Psyche. Die psychische Entwicklung der jungen Athleten wurde beeinträchtigt, und sie litten noch Jahre nach der Dopingperiode im Erwachsenenalter an Spätfolgen und Folgeerkrankungen.

**Methode:**

In der Studie wurden drei Gruppen verglichen: Leistungssportler mit Doping (I), Leistungssportler ohne Doping (II) und Personen, die keinen Sport treiben (III). Es wurden somatische und psychische Erkrankungen analysiert, um die negativen Auswirkungen des Dopings in der sensitivsten Entwicklungsphase im Jugendalter zu ermitteln. Bei den Teilnehmenden wurde eine Anamnese erhoben und sie erhielten einen Fragebogen mit standardisierten psychologischen Tests zum Ausfüllen.

**Ergebnisse:**

Die Dopingkohorte wies eine höhere Rate an somatischen Erkrankungen, psychischen Störungen sowie sozialen und beruflichen Schwierigkeiten auf. Die Unterschiede waren geschlechtsspezifisch, wobei Männer häufiger an Leberfunktionsstörungen, Depressionen, Tumorerkrankungen und arbeitsplatzbezogenen Problemen litten. Die Dopinggruppe berichtete über mehr emotionale und körperliche Vernachlässigung in der Kindheit. Sie erwies sich als weniger optimistisch, dafür pessimistischer, nahm weniger soziale Unterstützung wahr und hatte mehr Depressionen. In der Studie wurden weniger Extraversion und mehr Neurotizismus festgestellt. Posttraumatische Belastungsstörungen (PTSD) traten bei einer kleinen Anzahl von Teilnehmenden der Dopinggruppe auf. Doping wird mit psychiatrischen Variablen in Verbindung gebracht. Prädiktoren waren die Subskala zur Identifizierung von Gefühlen der Toronto Alexithymia Scale 20 (TAS-20), das Kohärenzgefühl und das Beck Depression Inventory 2 (BDI-II) sowie der Beck Depression Inventory (BDI).

**Schlussfolgerung:**

Die körperlichen und psychosozialen Auswirkungen deuten auf einen Zusammenhang mit der Anwendung von Dopingsubstanzen hin, könnten aber nicht nur auf die Nebenwirkungen dieser Substanzen zurückzuführen sein, sondern auch durch das System, welches großen psychischen Druck und Stress auf die sehr sensible Entwicklungsphase der Adoleszenz ausübte, bedingt sein.

## Introduction

Doping is a major problem in sporting competition, both at national and international levels. Individual sports and sports with high financial pressure are particularly affected; however, substances are not only used to improve performance in adulthood, but also in childhood and adolescent development, where targeted intake of anabolic steroids, for example, is/was used to change the body and improve performance; however, the use of these substances causes a lot of damage to various organ systems. Not only physical, but also psychological and social consequences are to be expected.

## Background

Doping includes the use of agents, e.g., anabolic-androgenic steroids (AAS) to enhance athletic performance in the prestigious success of medal winning athletes as part of the competition with western societies [8].

The German Democratic Republic (GDR) introduced the governmental doping plan 14.25 with a mandatory doping program. Approximately 15,000 athletes were exposed to these compulsory and harmful procedures [8]. The respective substances selectively increase physical performance but have serious somatic and psychological side effects when used in high dosages and for longer times [5]. Doping substances were applied during adolescence in a very vulnerable period. This was embedded in a traumatizing system separating young athletes from their homes and exposing them to the harshness and potential recklessness of trainers. Substances were administered to athletes without their consent or knowledge but under defined control conditions. Because the young athletes trusted their coaches and medical teams, they took the oral medications, had intramuscular injection and/or infusions [8].

Some adolescent athletes trained with comparable workloads to seniors. This could only be compensated by taking the abovementioned substances and ended in young age injuries that abruptly stopped the athletic career. Thereafter, athletes did not receive any support or training, and moral guilt was suggested by officials, because they had let their “team mates down” [8]. Thereby, AAS use resulted in physical injuries, secondary organic diseases, that could have an impact on the following generation, and in psychological damage such as depression, anxiety disorder, increased aggressiveness, and disturbance of identity.

## Somatic effects

The application of AAS results in, e.g., cardiac, (neuro)endocrine, hepatic and musculoskeletal disorders [48]. Cardiac effects are the development of cardiomyopathy, arrhythmia, and hypertension. The enlargement of the ventricle (cardiomyopathy) leads to reduced ejection fraction due to decreased contractility. For AAS users a reduction in electrical stability was described [51] as well as abnormal cardiac autonomic tone regulation [40] and abnormal ventricular repolarization [40] inducing arrhythmia. Consumption of AAS also generates dyslipidemia with increased levels of Low-Density-Lipoprotein (LDL) and a decrease of High-Density-Lipoprotein (HDL) with an increased risk of atherosclerosis [24]. This explains frequent cerebrovascular and cardiac infarctions, especially in younger athletes.

Other side effects of AAS are known to cause hepatotoxicity with cholestasis [60], peliosis hepatis [60] and hepatic cancer [23]. Furthermore, neuroendocrinological disorders are recorded. Substances induce changes in physical appearance such as hirsutism and gynecomastia causing psychologic problems missing a psychosexual identity. The reduction of pituitary secretion of Luteinizing Hormone (LH) and Follicle-Stimulating Hormone (FSH) ended in a suppression of spermatogenesis, hypogonadism and infertility in men, and virilizing effects, dysregulation of menstruation and infertility in women [48].

Growth hormones, such as somatotropin (somatropin in the GDR), administered in doses 10 times higher than the therapeutic dose [4] may cause fluid retention, headache, and hypertension [4]. Further undesired effects may include acromegaly, cardiomyopathy, insulin resistance/diabetes and renal failure as well as osteoarthritis [4]. Erythropoetins increase plasma viscosity, increasing the risk of thrombosis, cardiovascular events, and stroke [4].

## Psychological effects

Psychological effects of doping pertain to the athletes’ emotional regulation, with anxiety and anger often being the consequence of low frustration tolerance [45]. Moreover, especially females tend to doubt performance, disrupted concentration, and somatic anxiety. Negative patterns of perfectionism were related to higher levels of cognitive anxiety and lower levels of self-confidence [37]. Eating disorders and distorted body images were also observed [47]. The prevalence of psychiatric symptoms in athletes with a record of doping, the figures vary, but the indication for a higher risk of psychopathologic manifestations inherent to doping is robust. The incidence of mood disorders (e.g., depression) was reported to be 22% in athletes [45] with doping compared to 10% in the general population [33]. Likewise, the prevalence rates of social anxiety, panic disorder and generalized anxiety disorder were 15%, 4.5% and 7.1%, respectively, among doped athletes [25], compared to 2.7%, 2.0% and 2.2% respectively, in the general population [33].

Psychopathologic sequelae of doping during childhood and adolescence are manifold similar to posttraumatic situations. This could suggest that athletes are at a higher risk of posttraumatic symptomatology, e.g., posttraumatic stress disorder (PTSD). Freyberger et al. [17] reported a prevalence of 29.9% in (state doped) GDR athletes compared to a lifetime prevalence of 8% in PTSD in the general population [34]. This supports the potential of the GDR’s doping system to have a traumatizing character.

The present study aims to investigate the physical as well as the mental sequelae of the state controlled doping in former athletes in the former GDR. We hypothesize trauma-related developmental pathogenesis of the psychopathologic manifestations, with their roots in childhood. We therefore analyzed childhood trauma along with personality traits and psychopathology as well as physical complaints and compared those variables between a group of doped athletes and two control groups non-doped athletes and sports-inactive individuals.

## Methods

A total of 159 patients were included in the study after giving informed consent and then divided into three groups. The first group (I) (*n* = 55) practiced competitive sport during adolescence and received AAS in the former GDR. The second group (II) (*n* = 50) intensively exercised sport without taking any chemical substances and the third group (III) (*n* = 54) did not engage in sport.

We interviewed patients who presented as outpatients for an orthopedic examination. We asked them for organic (musculoskeletal, cardiac, endocrinological, urogynecological, tumor), social (professional and family problems) and psychological (aggression, insomnia, anxiety, depression, fatigue, substance abuse, lethargy, perfectionism) features of the disorders.

In addition, the participants in this study filled in the following questionnaire of psychometric questionnaires. The study was approved by the institutional review board (IRB) of the Medical University (No. 128/18).

### PDS

The posttraumatic stress diagnostic scale (PDS) assesses symptoms of PTSD identifying potentially traumatic events [16]. The PDS has high internal consistency (coefficient alpha = 0.92).

### Neo FFI

The Neo Five-Factor Inventory (NeoFFI) [11] assesses the 5‑factor model of personality using 5 subscales (neuroticism, extraversion, openness, agreeableness, conscientiousness), each rated on a 5-point Likert scale. The so-called big five represent empirical observations of essential factors for personality description [14].

### TAS 20

The Toronto alexithymia scale 20 (TAS 20) measures difficulties describing feelings, identifying feelings and externally oriented thinking on a 5-point Likert scale [2] with good psychometric properties.

### Social support

The social support questionnaire assesses the perception and anticipation to receive support from others [19]. The used version consists of 14 items and is based on a 5-point Likert scale with good psychometric properties.

### ASI

The anxiety sensitivity index (ASI) comprises 18 items and the dimensions: somatic complaints, social concerns, and cognitive concerns. The questionnaire is a valid and reliable measure of anxiety sensitivity [32].

### SOC-L9

The Sense of Coherence Scale-Leipzig short scale (SOC-L9) [39] is a 9-item version of the coherence scale. It is valid and reliable to measure the sense of coherence. The construct comprises comprehensibility, manageability, meaningfulness, and self-confidence correlated with well-being.

### LOT

The life orientation test (LOT) [22] comprises the subscales pessimism and optimism correlated to psychological disorders (e.g., depression) in the general population.

### BDI-II

The Beck depression inventory 2 (BDI-II) is a widespread measure of depression [3]. It is assigned good psychometric properties and functions as a self-rating measure.

### CTQ

The childhood trauma questionnaire (CTQ) assesses in the dimensions physical, emotional, and sexual abuse as well as physical and emotional neglect. It is reliable and valid for this purpose and considered the gold standard in its field [35].

### Equality, diversity and inclusion statement

Our study included all identified cases of doping that presented to our outpatient clinic as part of a specialist review. No distinctions were made between gender, age and ethnicity; however, it must be mentioned that state doping in the GDR selected the cohort.

The team of authors consists of female and male contributors from the fields of orthopedics, psychosomatics and epidemiology.

With respect to the analysis, care was taken when planning the number of cases to ensure a similar gender ratio in the control groups as in the doping group.

The patient and public were not involved in the design, conduct, reporting or dissemination plans of the research.

## Results

A total of 159 persons were included in this study. The mean age of the doping group (group I, *N* = 55) was 55.3 years, in the sports group (group II, *N* = 50) 54.8 years and in the no sports group (group III, *N* = 54) 56.4 years. There were 30 females and 25 males in group I, 20 females and 30 males in group II, and 32 females and 22 males in group III. The sports disciplines group I was divided into endurance sports (*N* = 19), weight training (*N* = 12), ball sport (*N* = 10), martial sport (*N* = 9) and others (N = 5). Group II showed a similar distribution with endurance sport (*N* = 23), weight training (*N* = 11), ball sport (*N* = 8), others (*N* = 7), only martial sport (*N* = 1) was lower. In group I, 41 individuals reported use of oral turinabol, 28 use of dynvital, 15 used athletovit (both supplements, which were mixed with mestanolone) and 14 disclosed to have received injections with doping substances. Of the participants 30 claimed further substances or therapies, such as mixed drinks, powder drinks, protein and vitamin supplements, blood exchange and radiation therapy.

## Physical side effects

The most musculoskeletal diseases reported in group I were spinal syndromes (*N* = 42, 76.4%), arthropathies (*N* = 33, 60%), osteopathies (*N* = 21, 38.2%) and chronic myopathies (*N* = 34, 61.8%). In group II, 23 (46.9%) persons complained about spinal syndromes, 20 (40.8%) about arthropathies, 6 (12.2%) about osteopathies and 5 (10.2%) about chronic myopathies. In group III, there was an increased rate of spinal syndromes (*N* = 26, 49.1%). The rate of arthropathies (*N* = 17, 32.1%), osteopathies (*N* = 2, 3.8%) and myopathies (*N* = 5, 9.4%) were lower. In summary, the doping cohort had a significantly higher rate of all musculoskeletal disorders than the no-sport group (spinal syndromes odds ratio, OR = 3.4, *p* = 0.005, arthropathies OR = 3.2, *p* = 0.004, osteopathies OR = 15.8, *p* < 0.0005, myopathies OR = 15.5, *p* < 0.0005). There were also significant differences for spinal syndromes (OR = 3.7, *p* = 0.002), osteopathies (OR = 4.4, *p* = 0.003) and myopathies (OR = 14.2, *p* < 0.0005) between the doping and the sport group.

For other somatic disorders, we defined cardiac, endocrinological, urogynecological and tumor entities. Group I had a higher rate for arrhythmia than group II (34.5% vs. 8.2%, OR = 5.9, *p* = 0.002) and group III (34.5% vs. 8.2%, OR = 6.5, *p* = 0.001). Group I also had a higher probability for arterial hypertension than group II (38.2% vs. 17%, OR = 3.0, *p* = 0.018). Hepatopathy was more frequent in group I (25.5%) than in group II (4% or 8.2%) and group III (1% or 1.9%). There was a higher risk for hepatopathy for doped individuals compared to group II (OR = 3.8, *p* = 0.036) and group III (OR = 17.8, *p* < 0.0005).

Tumor diseases showed a higher risk for group I vs. group II (OR = 4.0, *p* = 0.015) and group III (OR = 4.3, *p* = 0.008).

With respect to the urogynecological diseases, in group I more cases of dysmenorrhea than in group II (27.3% vs. 8.2%, OR = 4.2, *p* = 0.02) were shown. There were no differences for other urogynecological diseases, such as late age of menarche, hormonal imbalance, complication encountered in pregnancy, abortion and urogynecological tumor between all groups.

## Social and professional consequences

Difficulties in profession, changes of profession, occupational disabilities, sick leave > 10 days, not being able to work in a team showed differences between group I and other groups. No differences between groups were observed for extended education. Especially difficulties in profession increased in group I vs. group II (OR = 114.8, *p* < 0.0005) and vs. group III (OR = 159.4, *p* < 0.0005). Sick leave > 10 days/year was increased in group I vs. group II (OR = 55.0, *p* < 0.0005).

For psychiatric disorders, we recognized a higher risk for depression (OR = 9.2, *p* < 0.0005) and sleeping disorders (OR = 3.9, *p* = 0.002) in group I vs. group II and vs. group III for depression (OR = 13.7, *p* < 0.0005), sleeping disorders (OR = 3.8, *p* = 0.001), anxiety disorder (OR = 17.8, *p* = 0.000).

Differences in psychosocial aspects with respect to gender were identified. Female participants in group I were at a higher risk for the categories difficulties in profession, change of profession, occupational disability, sick leave > 10 days/year, not being able to work in a team, and difficulties in close relationships. Although all these items were increased for the male participants. The risk in females was further increased for difficulties in profession (males OR = 287.5 vs. females OR = 90.0, *p* < 0.0005), sick leave > 10 days/year (males OR = 40.1 vs. females OR = 12.3, *p* < 0.0005) and not being able to work in a team (males OR = 46.2, *p* < 0.0005 vs. females OR = 5.1, *p* = 0.012).

Significant increases in females in group I for musculoskeletal diseases (spinal syndromes OR = 4.0, *p* = 0.010, arthropathies OR = 2.9, *p* = 0.035, osteopathies OR = 6.9, *p* = 0.002, myopathies OR = 16.2, *p* < 0.0005) were observed which were not found for male individuals.

Diseases such as arrhythmias were found to be increased in both males and females. Furthermore, an increased risk of liver disease was found in males (OR = 16.0, *p* < 0.0005), whereas no significance could even be found in females. In females, there was a difference between group I and the others for dysmenorrhea (OR = 3.0, *P* = 0.030) and late menarche (OR = 5.1, *p* = 0.012). Here, only females showed increased rates of depression in group I and the other groups (OR = 5.0, *p* = 0.002). Whereas for males these differences were observed for sleep disturbance, aggression, anxiety disorder and depression. Depression showed an increased risk in comparison to the female participants (male OR = 52.1, *p* < 0.0005 vs. female OR 5.0, *p* = 0.002). Males also showed increased risk for tumors in group I (OR = 9.5, *p* = 0.005) and pediatric diseases, e.g. orthopedic, autoimmune, dermatologic, psychological, endocrinological (OR = 4.2, *p* = 0.029).

There were no differences between group II and group III but for arterial hypertension (OR = 2.2, *p* = 0.043).

## Psychological effects

In group I individuals claimed more childhood emotional and physical neglect, to be less optimistic and more pessimistic, to perceive less social support and to be far more depressive than the other groups. Moreover, group I displayed less extraversion and more neuroticism. Additionally, the history of doping was linked to anxiety sensitivity with respect to all subscales (Table 1).

**Table 1:** Comparison of the variables of interest between the groups with doping experience, sports persons and no-sports persons

	1) Doping	2) Sports	3) No sports	F	p	Bonferroni
CTQ (emotional abuse)	7.87 (3.86)	6.44 (2.41)	7.48 (3.53)	2.44	0.9	–
CTQ (physical abuse)	6.48 (2.63)	5.69 (1.49)	6.17 (2.61)	1.49	0.2	–
CTQ (sexual abuse)	5.93 (2.21)	5.47 (2.26)	5.53 (1.68)	0.76	0.5	–
CTQ (emotional neglect)	10.87 (5.12)	8.10 (4.12)	10.17 (4.86)	4.61	*0.01*	*1 vs. 2*
CTQ (physical neglect)	8.43 (3.07)	7.33 (2.69)	6.69 (2.28)	5.59	*0.005*	*1 vs. 3*
TAS-20 (describing feelings)	2.69 (0.46)	2.52 (1.40)	2.44 (0.54)	1.13	0.3	–
TAS-20 (identify feelings)	2.43 (0.84)	1.59 (0.59)	1.64 (0.55)	25.10	0.0000	*1 vs. 3*
TAS-20 (external oriented thinking)	3.08 (0.38)	3.01 (0.44)	3.14 (0.40)	1.28	0.3	–
LOT (pessimism)	2.69 (0.77)	2.18 (0.78)	2.17 (0.71)	8.07	*0.000*	*1 vs. 2* *+* *3*
LOT (optimism)	3.58 (0.86)	4.14 (0.80)	4.06 (0.79)	7.11	*0.001*	*1 vs. 2* *+* *3*
Social support	4.00 (0.66)	4.55 (0.43)	4.40 (0.52)	14.02	*0.000*	*1 vs. 2* *+* *3*
BDI-II (depression)	16.88 (11.22)	5.21 (8.78)	5.65 (5.76)	28.57	*0.0000*	*1 vs. 2* *+* *3*
NEO-FFI (neuroticism)	2.73 (0.63)	2.21 (0.49)	2.31 (0.56)	12.46	*0.0000*	*1 vs. 2* *+* *3*
NEO-FFI (extraversion)	3.02 (0.68)	3.59 (0.56)	3.44 (0.63)	11.33	*0.000*	*1 vs. 2* *+* *3*
NEO-FFI (openness)	3.25 (0.61)	3.22 (0.48)	3.43 (0.57)	2.14	0.1	–
NEO-FFI (agreeableness)	3.50 (0.41)	3.59 (0.48)	3.66 (0.55)	1.5	0.2	–
NEO-FFI (conscientiousness)	4.03 (0.55)	4.21 (0.48)	4.19 (0.49)	2.07	0.1	–
Sense of coherence	44.16 (8.69)	51.04 (5.41)	47.63 (7.43)	10.81	*0.000*	*1 vs 2*
ASI (physical concerns)	1.80 (0.79)	1.32 (0.68)	1.23 (0.64)	10.03	*0.000*	*1 vs. 2* *+* *3*
ASI (mental incapacitation concerns)	1.68 (0.88)	1.03 (0.72)	1.11 (0.65)	11.62	*0.000*	*1 vs. 2* *+* *3*
ASI (social concerns)	1.70 (0.85)	1.29 (0.71)	1.26 (0.67)	5.66	*0.004*	*1 vs. 2* *+* *3*

The table shows the mean ± SD (standard deviation) of psychological assessment of the three groups, as well as the variance-quotient (*F*) and the significance level (*p* < 0.05). Bonferroni: adjustment for multiple comparison.

*CTQ* childhood trauma questionnaire, *TAS* Toronto alexithymia scale, *LOT* life orientation test, *BDI* Beck depression inventory, *FFI* 5 factor inventory, *ASI* anxiety sensitivity index

Regarding the A1 traumas (a person was exposed to actual or threatened death, serious injury, sexual violence), no significant results showed for the experience of an accident (*χ*^2^ = 2.0, *p* = 0.4), violence within families (*χ*^2^ = 4.17, *p* = 0.1), sexual abuse within family (*χ*^2^ = 0.17, *p* = 0.09), or combat (*χ*^2^ = 2.94, *p* = 0.3) and imprisonment (*χ*^2^ = 1.88, *p* = 0.4); however, attacks by a stranger and sexual assault by a stranger were more frequent in group I (Table 2). The PTSD only occurred in group I (*n* = 3) but was not significant compared to other groups (Table 2).

**Table 2:** Cross-tabulation of PTSD, its criterion A1 and the categories doping, sports and no sports (only significant results are shown)

	Natural catastrophe	No natural catastrophe	Assault by a stranger	No assault by a stranger	Sexual assault by a stranger	No sexual assault by a stranger	PTSD	No PTSD
Doping (53)	7 (13.21%)	46 (86.79%)	17 (32.08%)	36 (67.92%)	7 (13.21%)	46 (86.79%)	3 (5.66%)	52 (94.34%)
Sports (49)	13 (26.53%)	36 (73.47%)	7 (14.29%)	42 (85.71%)	0 (0%)	49 (100%)	0 (0%)	49 (100%)
No sports (54)	5 (9.26%)	49 (90.74%)	2 (3.70%)	52 (96.30%)	4 (7.41%)	50 (92.59%)	0 (0%)	54 (100%)
*χ*^2^ ≤ *p*	6.17 ≤ 0.05		15.79 ≤ 0.000		6.79 ≤ 0.03		5.78 ≤ 0.06	

*PTSD* posttraumatic stress disorder

Using binary regression analysis (Table 3), we aimed to delineate the closest relation of the psychiatric variables associated with doping to that category. The respective predictors were the subscale identifying feelings of the TAS-20 (*p* = 0.000), the sense of coherence and the BDI‑II (*p* = 0.002) as well as the BDI (*p* = 0.02).

**Table 3:** Binary regression analysis; dependent variable: groups with doping and without doping (sports persons and no sports persons)

	B	SE	Wald	Df	p	Exp (B)	CI lower	CI upper
CTQ (physical neglect)	−0.09	0.13	0.50	1.00	0.48	0.91	0.71	1.18
CTQ (emotional neglect)	0.03	0.07	0.13	1.00	0.72	1.03	0.89	1.18
Gender	0.69	0.58	1.42	1.00	0.23	1.99	0.64	6.20
LOT (pessimism)	−0.14	0.44	0.10	1.00	0.75	0.87	0.37	2.05
LOT (optimism)	0.19	0.49	0.15	1.00	0.70	1.21	0.46	3.16
Social support	1.29	0.56	5.34	1.00	*0.02*	3.64	1.22	10.92
BDI-II (depression)	−0.12	0.05	7.50	1.00	*0.01*	0.88	0.81	0.97
NEO-FFI (neuroticism)	0.23	0.70	0.11	1,00	0.75	1.25	0.32	4.93
NEO-FFI (extraversion)	0.47	0.50	0.89	1.00	0.34	1.60	0.60	4.22
Sense of coherence	−0.24	0.08	9.44	1.00	*0.002*	0.78	0.67	0.92
ASI (physical concerns)	0.01	0.73	0.00	1.00	0.99	1.01	0.24	4.27
ASI (mental incapacitation concerns)	−0.14	0.67	0.04	1.00	0.83	0.87	0.23	3.23
ASI (social concerns)	0.18	0.62	0.09	1.00	0.77	1.20	0.36	4.04
TAS-20 (identify feelings)	−2.10	0.60	12.19	1.00	*0.000*	0.12	0.04	0.40

Binary regression analysis: *B* regression coefficient, *SE* standard error, *Wald* Wald statistics with dregrees of freedom (*Df*) and calculated *p*-value (the figures in italics show the significant values (*p* < 0.05)), *Exp* *(B)* effect strength equates to Odds-Ratio, *CI* 95%-confidence intervall lower and upper for Exp (B), *CTQ* childhood trauma questionnaire, *LOT* life orientation test, *BDI* Beck depression inventory, *FFI* 5-factor inventory, *ASI* anxiety sensitivity index, *TAS* Toronto alexithymia scale

## Discussion

In the state doping of the GDR, athletes were administered steroid-containing, modifying substances without their knowledge. After > 30 years, late effects of AAS show up on physical and psychosocial levels. To our knowledge, this is the first study to compare the long-term effects of systematic doping during adolescence.

Our results analyze the risk for the abovementioned organic and structural damage after anabolic abuse in competitive sports.

With a threefold (doped vs. non-sport) and a fourfold (doped vs. sport), respectively, increased risk for spinal syndromes and arthropathies, our data show a slightly higher risk compared to a study by Horn et al. [28] in football players after professional career with a 2-fold increased risk after AAS use.

Osteopathies and myopathies showed an increased risk to occur after doping, which can explained by a change in the collagen structure [12] and changes in tendon elasticity [42]. Bradytrophic tissues, such as tendons cannot compensate for the steroid-induced force increase [13]. Differences in arthropathies were not found between doping and sports groups, which may be due to increased training intensity with a general damage to articular cartilage in competitive athletes.

The most commonly observed arrhythmias occurring in athletes are atrial fibrillation, ventricular fibrillation, ventricular tachycardia, and supraventricular and ventricular ectopy [27]. Cardiomyopathies were frequently observed in other studies after AAS use which we did not observe in our cohort. This effect occurs during or shortly after a competitive sports career or AAS use. Over time, the myocardium adapts to the new conditions after a reduction of the load [46]. Thereby, concentric left ventricular hypertrophy may be evident years after discontinuation of AAS; however, ventricular hypertrophy is considered to be caused by excessive training and not only by AAS abuse [57].

The liver dysfunction has a significantly increased risk in our study. Direct hepatotoxicity of AAS has been described leading to cholestasis, peliosis hepatis and liver adenomas with ultrastructural damage via oxidative stress [49]. Moreover, there is an indirect effect inhibition of steroid metabolism to cholesterol storage to steatosis hepatis, which may be reversed after discontinuation of AAS [56]. Sometimes the adenomas developed into liver carcinomas [21]. Maybe the reason for liver damage is a rate of 80% metabolization process of androgens by the liver which accounts for the gender effects. A connection between AAS intake and hepatocellular carcinomas was described in other studies. The intracellular androgen receptor is not only found in reproductive organs but also in bone, muscle, liver, brain and kidney cells. Binding to the receptor initiates a cascade that ultimately leads to altered gene expression [20]. In the doping group, there was a fourfold increased risk of tumors and male athletes had a further increase. Why female doped athletes have neither an increased risk for liver dysfunction nor for tumor diseases remains unexplained and requires further studies analyzing gender-specific effects.

Urogynecological complications, such as dysmenorrhea and late onset of menarche can be explained by the intervention in the female hormonal balance. Other effects include hirsutism, irreversible deepening of voice, secondary amenorrhea, anovulation, and ultimately infertility [59].

The group of doped competitive athletes differ in family and work-related problems from those of athletes and non-athletes. Difficulties at work are frequently reported. Many former athletes with AAS have a disruptive professional career with extended time of education, unemployment, as well as frequent retraining leading to occupational disability due to physical and psychological disorders. Family situations are also characterized by difficulties in partnerships. The reduced adaptability is often pronounced and not being able to work in a team is also described. Differences in gender could be linked to diverse psychological pathologies, such as depression, anxiety disorders, aggression, sleeping disorders, and non-team ability. Males show a 46-fold increased risk compared to the female athletes with a 5-fold increased risk. In female athletes, only depression was found to be a significant psychological disorder. Extended sick leave (sick days > 10/year) was found for male athletes, whereas regarding occupational disability the female doping group again showed an increased risk. This may suggest that former female athletes with AAS use are less likely to call in sick, but thereby exceed their physical and mental capacity. This was already crucial during their athletic career, and therefore they might be more likely to suffer from occupational disability as they no longer have any further compensatory mechanisms.

The consequences of state-controlled doping were investigated with respect to the history of childhood trauma, PTSD, and current psychopathology. As prior findings on the effects of doping, we found an increased burden of pessimism and depression, along with a decreased comfort through social support and a weaker sense of coherence; however, depression turned out to be the psychological symptom most closely linked to the doping experience. More interestingly, on the personality level this study reveals a relationship between the experience of state-driven doping and alexythymia, the sense of coherence and childhood trauma.

The doped cohort displayed more difficulty identifying feelings (i.e., alexithymia), more neuroticism and less extraversion, as well as higher levels of anxiety sensitivity and a weakened sense of coherence. The experiences of physical and emotional neglect in childhood were significantly more pronounced in the doped than in the remaining categories. As to PTSD, no significant difference showed, although the respective A1 criterion (i.e., having experienced a certain trauma) was clearly linked to the history of doping with the traumas indicated being of interpersonal kinds, namely physical and sexual offence by nonmembers of the family.

Hence, our results add to the literature proposing the link between doping experiences and psychological disorders. Yet, this study develops a more comprehensive picture as the developmental perspective is reconstructed suggesting that the vulnerability for the later distress is conveyed by childhood trauma, which is also reflected in the disclosure of type 1 interpersonal and sexual traumas by doped individuals and mediated by alexithymic traits as well as the sense of coherence and poor social support. Prior trauma, especially in childhood is known to be linked to adult alexithymia [55] as well as to adult psychopathology [58]. Alexithymia represents a relatively stable personality style including the reduced ability to realize, identify and express one’s emotions [54]. The deficient interpersonal recognition and communication of emotional states may lead to rejection by the social counterpart, which has at least been shown in psychotherapy [44]. The psychological disposition of alexithymia is also linked to physical disease, such as hypertension [10], intensity of chronic pain [41], heart rate regulation [6], dysmenorrhea [15], myopathy [1] and hepatopathy [9]. This pattern of clinical associations is represented in the doping group of our study. The mechanism behind these associations has been described as higher symptom reporting and perception, somatization as well as a tendency towards unhealthy lifestyles [9]. Considering these implications of alexithymia, its association with childhood trauma is perspicuous, because childhood trauma is linked to several physical conditions, such as back pain [38], arthritis [18] and myopathy [18].

However, the connection between childhood trauma and arrhythmias, hypertension, and hepatopathy has not been reported previously. Dysmenorrhea was also more frequent in the doped individuals, although this may not be a posttraumatic effect [29]; however, age at menarche is reflective of childhood trauma [7] and was linked to doping in the present study.

Most athletes start sport in childhood or adolescence [53], where they may experience injury and competition. Such stressors may precipitate psychiatric disorders, especially depression, anxiety and attention deficit hyperactivity disorder (ADHD) [53]; however, depression which we found to be the most likely symptom in the aftermath of doping, is not overrepresented in elite athletes [53], suggesting our finding to be caused by the doping experience rather than by the athletic status itself. Apart from psychological sequelae, such as alexithymia, doping is linked to bodily changes in women and men, which, when encountered in childhood and/or adolescence, may critically interfere with the consolidation of identity and with the maturation of the psyche. Effects are acne, hirsutism, amenorrhea in females, as well as loss of libido and gynecomastia in males, or hospitalization because of e.g. liver damage independently of the athlete’s gender [52].

The present study revealed lower levels of the sense of coherence in doped athletes, which may reflect the destabilizing influence of the doping experience on the behavioral, cognitive and motivational resistance [43]. The sense of coherence (SOC) comprises opinion forming, which is a constituting element of resilience, and typically hampered by posttraumatic experiences [50]. Thus, reduced SOC may be an important factor within the cascade of physical, psychic and psychosomatic changes which labilize the formerly doped individuals of the present study.

Apart from the harmful effects of excessive training, the role of interpersonal trauma must be considered. In this study, 32% of the participants with an experience of state-driven doping reported the experience of an interpersonal assault by a stranger (i.e., not a member of the family) and 13.2% reported a sexual assault by a stranger, comparing to 4.9% in the general population [36]. This finding indicates that the trauma is associated with doping, and that those conditions effectuated a lack of emotional care and attention. In addition, Johnson et al. [30] reported the prediction of restricted cardiovascular health by childhood neglect, suggesting a pathway from childhood trauma to heart disease, a finding possibly reflected in the present results.

That aside, the toxicity of doping agents of course may contribute directly to physical restriction and disease. The cardiovascular system, for example, is believed to suffer damage through atherosclerosis, thrombosis, vasospasm or direct injury of the endothelium [26]. Apart from such deleterious effects on the cardiac system, AAS induce a variety of hormonal dysbalances to the effect of a systematically compromised gender-related health. The hormonal cascade of the Hypothalamic-Pituitary-Adrenal (HPA) axis and the ovarian function lose their balance and set the stage for virilization and organ dysfunction. Training itself, however, may have an effect on the hormonal regulation and the onset of puberty [31]. The finding [7] of a dose-dependent influence of childhood abuse, however, on the age at menarche, corresponds well to the present finding of childhood trauma in the doped cohort.

Hence [5], there are risks beyond being caught that come along with doping. Awareness for the hazardous potential should be raised among athletes and their trainers as well, who represent an important influence and source of information for athletes and, at the same time, might be at risk of damaging their fosterlings.

Despite its piloting character, the present study has a few caveats, as it is cross-sectional and retrospective in nature, and relies on a restricted sample; however, the cohorts of this study may serve as an admonishing example to athletes and sports officials in present and future times.

## Abbreviations

AAS: Anabolic-androgenic steroids
ADHD: Attention deficit hyperactivity disorder
FSH: Follicle-stimulating hormone
GDR: German Democratic Republic
HDL: High-density-lipoprotein
HPA: Hypothalamic-pituitary-adrenal
IRB: Institutional review board
LDL: Low-density-lipoprotein
LH: Luteinizing hormone
PTSD: Posttraumatic stress disorder

## Declarations

Patient and public involvement: patients and/or the public were not involved in the design, conduct, reporting or dissemination plans of this research. This study involves human participants and was approved by ethics committee of the Otto-von Guericke University Magdeburg (study 128/18). Participants gave informed consent to participate in the study before taking part.
